# Smartphone usage and overdependence risk among middle-aged and older adults: a cross-sectional study

**DOI:** 10.1186/s12889-024-17873-8

**Published:** 2024-02-09

**Authors:** Su Hyun Kim, Young Hoon Kim, Chang-Hyung Lee, YounYoung Lee

**Affiliations:** 1 Pukyong National University, Industry-University Cooperation Foundation, #45 Yongso-Ro, Nam-Gu, Busan, # 48513 Republic of Korea; 2 Department of Marine Sports, College of Information Technology and Convergence, Pukyong National University, #45 Youngso-Ro, Nam-Gu, Busan, # 48513 Republic of Korea; 3 Department of Physical Medicine & Rehabilitation, Research Institute for Convergence of Biomedical Science and Technology, Pusan National University Yangsan Hospital, Pusan National University School of Medicine, #20 Keumoro, Mulgeum, Yangsan, Gyeongnam # 50612 Republic of Korea; 4 Woon-Gok Liberal Arts Education College, Halla University, 28 Halla University-Gil, Wonju-Si, Gangwondo # 26404 Republic of Korea

**Keywords:** Smartphone, Smartphone overdependence, Content, Geriatrics

## Abstract

**Background:**

Previous studies have predominantly focused on smartphone overdependence among adolescents and young adults. However, as smartphone usage has recently surged among South Korean middle-aged and older adults, the risk of smartphone overdependence cannot be overlooked among this population. Therefore, this study was conducted to examine the smartphone usage pattern and the associated risk of overdependence in this specific age group.

**Methods:**

The data for individuals who aged 50 or older were extracted from the dataset of a nationwide survey, “The Survey on Smartphone Overdependence, 2021,” and the usage of each type of smartphone content and risk of smartphone overdependence among individuals in their 50 s and 60 s were investigated. Age-group-based differences in demographic characteristics, Smartphone Overdependence Scale scores, self-awareness of smartphone overdependence, digital literacy, and psychosocial factors were analyzed. Additionally, a multivariable logistic regression analysis was conducted to explore the factors associated with the potential-to-high risk of smartphone overdependence in both age groups.

**Results:**

Individuals in their 50s had significantly higher digital literacy, social relations, life satisfaction, and smartphone overdependence scores than those in their 60s, and the percentage of individuals in the high-risk group was also higher in the 50s age group. For both age groups, the most used content was “messenger,” “news,” and “movies/TV/videos,” whereas the least used content was e-learning, gambling, and adult content. The multivariable analysis indicated that, for individuals in their 50s, having a lower educational level was associated with significantly higher odds, whereas having a job and utilizing e-commerce-related contents on smartphone were associated with significantly lower odds of potential-to-high risk for smartphone overdependence. Concerning individuals in their 60s, having a lower educational level and using adult content or gambling were significantly associated with higher odds of potential-to-high risk for smartphone overdependence.

**Conclusion:**

This study reveals the risk of smartphone overdependence among middle-aged and older adults in South Korea as well as the associated risk factors. This will assist policymakers in developing policies for the appropriate use of smartphones by these age groups.

**Supplementary Information:**

The online version contains supplementary material available at 10.1186/s12889-024-17873-8.

## Background

Smartphone usage has increased exponentially and has become an essential part of life owing to the wide array of benefits it provides, such as comfort and accessibility [1]. Additionally, smartphones have proven to be beneficial as one can manage one’s lifestyle, physical activity, nutrition [2], and even metabolic diseases [3–5] using a smartphone. However, there have been concerns about excessive smartphone use harming physical and mental health, causing eye problems [6, 7], musculoskeletal diseases [8, 9], and psychological problems [10].

Problematic smartphone usage is called in various ways such as smartphone addiction, smartphone dependence, and smartphone overdependence, and there is no consensus on its diagnostic criteria. Smartphone addiction—the uncontrollable use of smartphones despite its various adverse effects—is not classified as a disease in the fifth edition of the Diagnostic and Statistical Manual of Mental Disorders (DSM-5). However, researchers believe that it should be considered a “behavioral” addiction within the Substance-Related and Addictive Disorders category in the DSM-5, like gambling disorder [11], because it induces cravings, preoccupation, uncontrollability, withdrawal, and problematic consequences in daily life [1, 12, 13]. In South Korea, the focus on the negative effects of smartphone usage has been increasing, and since 2004, the Ministry of Science and ICT and National Information Society Agency (NISA) have been administering an annual nationwide survey on internet and smartphone dependence to prevent addiction [14]. In this national statistical survey, the term “smartphone addiction” has been replaced with “smartphone overdependence” since 2016 [15]. They defined the concept of “smartphone overdependence” as a state in which the salience of smartphones increases, one’s control over smartphone use decreases, and one faces negative consequences owing to excessive smartphone use [15]. Smartphone overdependence risk is categorized into the following three groups: no-risk, potential-risk, and high-risk. The potential-risk group of smartphone overdependence is distinguished by a diminished level of control over smartphone usage and the onset of interpersonal conflicts or difficulties in executing daily responsibilities [15, 16]. Furthermore, the high-risk group is defined by a loss of control over smartphone usage and the experience of interpersonal conflicts or serious problems in performing daily responsibilities or maintaining health [15, 16]. This alteration, from addiction to overdependence, reflects a governmental policy that highlights individual capacity and choice, rather than pathologizing problematic smartphone use, acknowledging the indispensable role of digital devices in daily life [17].

Smartphone ownership has steadily increased from 83.3% in 2016 to 93.4% in 2021 [18]. Correspondingly, the proportion of individuals with a potential-to-high risk of smartphone overdependence has also increased—from 17.8% in 2016 [15] to 24.2% in 2021 [16]. In 2016, more than 96% of adults under 50 years of age owned a smartphone, and their smartphone ownership increased by 1%–3% over the last five years [18]. By contrast, smartphone ownership increased substantially among individuals over the age of 50, increasing from 89.2% to 98.4% for those in their 50 s and 60.3% to 91.7% for those in their 60 s [18]. This indicates that older adults have started embracing digital technologies, despite their tendency to slowly change their life patterns. As smartphones have become a necessity among all age groups, the risk of smartphone overdependence is no longer zero for any age group. However, previous studies on smartphone overdependence have predominantly focused on adolescents and young adults [6, 8–10, 12, 19, 20] and rarely on middle-aged or older adults, which highlights the need to investigate the potential-to-high risk of smartphone overdependence in this specific population.

In this rapidly evolving digital era, the digital divide—the disparity in access to digital technologies [21]—has emerged as an important social issue because it results in unequal opportunities and disadvantages for underserved populations. The concept of digital literacy, defined as “the ability to understand and use information in multiple formats from a wide range of sources when it is presented via computers” [22], has evolved to encompass an individual’s ability to locate, evaluate, and use digital information [21]. It signifies the capacity to access and effectively utilize digital resources, thus playing a pivotal role in bridging the digital divide. Digital literacy has been demonstrated to enhance individuals’ life satisfaction by influencing their smartphone use motives [23]. However, several studies have demonstrated a positive correlation between digital literacy and smartphone overdependence [24, 25], which requires further exploration.

In addition, the intricate relationship between smartphone usage and psychosocial factors is multifaceted. As individuals age, they experience loneliness and isolation owing to family structure or employment changes. In such circumstances, smartphones can prove useful, as they provide the opportunity to share information, stay in touch [26–28], and elevate overall life satisfaction [23]. Nonetheless, concerns regarding the negative effect of smartphone usage on mental health persist [25, 29].

Considering the dual nature of smartphone use encompassing both beneficial and detrimental aspects, determining ways to assist users in utilizing smartphones in a healthy manner, rather than simply suppressing their smartphone usage, is necessary. Therefore, to assist middle-aged and older adults, who are at risk of smartphone overdependence, in managing their smartphone usage, it is essential to determine their patterns of smartphone usage and dependency, as well as the associated factors contributing to the risk of overdependence. Furthermore, it can be inferred that the cumulative duration of smartphone usages by individuals in their 50 s and 60 s differs, as smartphone usage by individuals in their 60 s has increased by more than 30% over the last five years [18].Additionally, considering the mean retirement age of approximately 60 in South Korea [30, 31], it can be assumed that individuals in their 50 s and 60 s have different demographic and psychosocial attributes. Consequently, their smartphone usage and dependency may differ and should be independently investigated.

Therefore, this study aimed to investigate the usage of different types of smartphone content among middle-aged and older adults in South Korea, as well as the risk and associated demographic and psychosocial factors contributing to smartphone overdependence for both age groups.

## Method

### Data collection

This study used pre-existing survey data from “The Survey on Smartphone Overdependence, 2021” —a nationwide survey [16] administered in South Korea by the Ministry of Science and ICT and the NISA between September and November 2021. This survey targeted individuals aged 3—69 residing in South Korea who had used smartphones to access the internet at least once over the last month. The data collection was based on 372,373 districts identified in the “2019 Population and Housing Census” by the National Statistical Office. Sampling involved selecting survey districts from this census, resulting in a sample size of 10,000 households across 1,000 survey districts with proportional allocation. The effective sample comprised 10,000 households and 25,198 individuals within those households. The survey was administered through household visits and face-to-face interviews [16].

This anonymous dataset was accessed from a public data portal in South Korea (www.data.go.kr) and the data from individuals aged 50 years or older were selected. All variables included in this cross-sectional study were sourced from this extracted dataset.

### Variables used in the analysis

#### Demographic variables

Sex, age, household income, personal income, job status, educational level, and area of residence were included as the demographic variables. The area of residence was categorized into Metropolitan cities, Cities, and Small towns (Counties, Towns/Townships, and Eup/Myeon) according to South Korea’s administrative divisions.

#### Usage of different types of smartphone content

Twenty-five types of smartphone content were categorized into informative content (news, searches for study/work, searches for a hobby, searches for goods/services, searches for transportation, other web-surfing), entertainment content (games, movies/television/videos, music, radio/podcasts, e-books/webtoons/web stories), adult content and gambling, communication content (e-mail, messenger, social networking services, dating and meeting), e-commerce content (buying and selling goods), life management content (finance, life, health), and work and education content (online meetings/remote work, essential and private e-learning). The participants were asked to rate whether they use these smartphone contents and their frequency of use on a 7-point Likert scale (1 = rarely, 7 = very frequently). The scores were summed within each category to determine the degree to which each content category was used.

#### Smartphone overdependence

1. Smartphone Overdependence Scale

Participants’ smartphone overdependence was measured using the self-reported Smartphone Overdependence Scale for adults. This scale was developed and validated by the NISA in 2016 [32] by revising the pre-existing Korean Scale for Internet Addiction (K-scale) [33] and Smartphone Scale for Smartphone Addiction (S-scale) [34], designed for assessing internet and smartphone addiction in Korea. The scale comprises three categories and ten items—self-control failure (items 1–3), salience (items 4–7), and problematic consequence (items 8–10)—rated on a 4-point Likert scale (1 = not at all, 4 = very much). An additional file presents in-depth information concerning each item [see Additional file 1]. The cut-off levels for smartphone overdependence risk were determined based on the developers’ report [32]. Participants younger than 60 years who scored 29 or more and those aged 60 years or older who scored 28 or more were categorized into the “high-risk group.” Participants younger than 60 years who scored between 24 and 28 and those aged 60 years or older who scored between 24 and 27 were categorized into the “potential-risk group.” Finally, participants who scored less than 24 belonged to the “no-risk group”; they had good control over their smartphone usage [16, 32]. Cronbach’s alpha for the samples in this study was 0.907 (0.886 for the participants in their 50 s and 0.920 for those in their 60 s).

2) Self-awareness of smartphone overdependence

Self-awareness of smartphone overdependence was measured by asking participants to what extent they depended on their smartphones compared to others (1 = not dependent, 2 = less dependent than others, 3 = similar to others, 4 = more dependent than others, and 5 = much more dependent than others). In the analysis, responses of 1 or 2 were grouped as “less dependent than others,” and responses of 4 or 5 were grouped as “more dependent than others.”

#### Digital literacy, and psychosocial variables

Digital literacy and psychosocial variables, which have been assessed during the annual survey on smartphone overdependence since 2019, were included in the analysis to estimate its association with smartphone overdependence [35]. Digital literacy was measured using six items on a 4-point Likert scale—namely, “information retrieval,” “information evaluation,” “online societal awareness and engagement,” “digital content creation and editing,” “online privacy awareness,” and “educational and occupational use of online information”—developed by the NISA [35]. Cronbach’s alpha for this study’s samples was 0.866 (0.822 for the participants in their 50 s and 0.878 for those in their 60 s).

Psychosocial characteristics were assessed through two domains of social relations (three items) and life satisfaction (nine items) on a 4-point Likert scale, developed by the NISA [35]. An additional file provides more information about each item [see Additional file 1]. Cronbach’s alpha for this study’s sample was 0.820 (0.806 for the participants in their 50 s and 0.828 for those in their 60 s).

The total score of each category was used for analysis, and higher scores indicated higher levels of digital literacy, more social relations, and greater life satisfaction.

### Statistical analysis

Of the 25,198 participants in “The Survey on Smartphone Overdependence 2021,” the data from 10,001 individuals aged ≥ 50 were sourced. Categorical and continuous variables were expressed as numbers and percentages, and mean ± standard deviation. Chi-square tests were used to determine age-group-based differences in participants’ demographic characteristics, smartphone overdependence risk, and self-awareness of smartphone overdependence and to compare differences in participants’ demographics based on smartphone overdependence risk (no-risk *versus* potential-to-high risk). Additionally, this study determined how participants’ usage of smartphone content and their digital literacy and psychosocial characteristics differed based on age-group and smartphone overdependence risk using student’s t-test. Furthermore, a multivariable binary logistic regression analysis was conducted to define the odds ratios (ORs) classified in the “potential-to-high risk group of smartphone overdependence,” considering their demographic and psychosocial characteristics, digital literacy and smartphone content usage patterns as independent variables. All statistical analyses were performed using IBM SPSS software (version 27; IBM Corp., Armonk, NY, USA), and statistical significance was set at *p* < 0.05.

## Results

Table 1 presents the participants’ demographic and psychosocial characteristics and smartphone dependence based on their age group. Educational level, household income, job status, and area of residence significantly differed based on age group. Participants in their 50 s had significantly higher digital literacy (15.05), more social relations (8.63), and greater life satisfaction (20.87; *p* < 0.001) than those in their 60 s. Additionally, participants in their 50 s had significantly higher smartphone dependence (18.28, *p* < 0.001) than those in their 60 s (16.64), and scored significantly higher on all categories and items. An additional file presents the total and item scores in more detail [see Additional file 1]. Of the participants in their 50 s, 15.5% and 3.9% had potential- and high-risk smartphone overdependence, respectively, whereas that for participants in their 60 s was 13.1% and 3.1%, respectively. Participants’ self-awareness of smartphone dependence differed significantly based on age group.

**Table 1:** Participants’ characteristics based on age group

		N (%) or Mean ± SD		
		50–59 years (*N* = 4791)	60–69 years (*N* = 5210)	Total (*N* = 10001)
Sex	Male	2295 (47.9%)	2565 (49.2%)	4860 (48.6%)
	Female	2496 (52.1%)	2645 (50.8%)	5141 (51.4%)
Education^*^	< middle school graduates	96 (2.0%)	1311 (25.2%)	1407 (14.1%)
	High school graduates	2771 (57.8%)	3335 (64.0%)	6106 (61.1%)
	≥ University graduates	1924 (40.2%)	564 (10.8%)	2488 (24.9%)
Monthly household income^*^	< KRW 4 million	1331 (27.8%)	3431 (65.9%)	4762 (47.6%)
	KRW 4–6 million	2169 (45.3%)	1044 (20.0%)	3213 (32.1%)
	≥ KRW 6 million	1291 (26.9%)	735 (14.1%)	2026 (20.3%)
Monthly personal income^*a^	< KRW 3 million	1782 (46.0%)	2303 (65.5%)	4085 (40.8%)
	≥ KRW 3 million	2093 (54.0%)	1215 (34.5%)	3308 (33.1%)
Job status^*^	No	912 (19.0%)	1692 (32.5%)	2604 (26.0%)
	Yes	3879 (81.0%)	3518 (67.5%)	7397 (74.0%)
Area of residence^*^	Metropolitan	2235 (46.6%)	2147 (41.2%)	4382 (43.8%)
	City	1652 (34.5%)	1743 (33.5%)	3395 (33.9%)
	Small town	904 (18.9%)	1320 (25.3%)	2224 (22.2%)
Digital literacy^*^		15.05 ± 3.70	12.79 ± 4.12	13.87 ± 4.08
Psychosocial characteristics	Social relations^*^	8.63 ± 1.63	8.37 ± 1.67	8.50 ± 1.68
	Life satisfaction^*^	20.87 ± 3.16	20.13 ± 3.37	20.49 ± 3.29
Smartphone Overdependence Scale	Self-control failure^*^	6.18 ± 1.97	5.47 ± 2.12	5.81 ± 2.08
	Salience^*^	5.61 ± 1.99	5.10 ± 2.07	5.34 ± 2.05
	Problematic consequence^*^	6.49 ± 2.13	6.08 ± 2.09	6.28 ± 2.12
Risk of smartphone overdependence^*b^	No-risk	3862 (80.6%)	4368 (83.8%)	8230 (82.3%)
	Potential- risk	742 (15.5%)	681 (13.1%)	1423 (14.2%)
	High-risk	187 (3.9%)	161 (3.1%)	348 (3.5%)
Self-awareness of smartphone dependence^*^	Less dependent	1523 (31.8%)	2960 (56.8%)	4483 (44.8%)
	Similar to others	2706 (56.5%)	1858 (35.7%)	4564 (45.6%)
	More dependent	562 (11.7%)	392 (7.5%)	954 (9.5%)

^*^p < 0.001 by χ^2^ test or student’s t-test between age groups

^a^Discrepancy in the total numbers of participants is attributed to missing data (2608 cases)

^b^based on the Smartphone Overdependence Scale

**Fig. 1:**
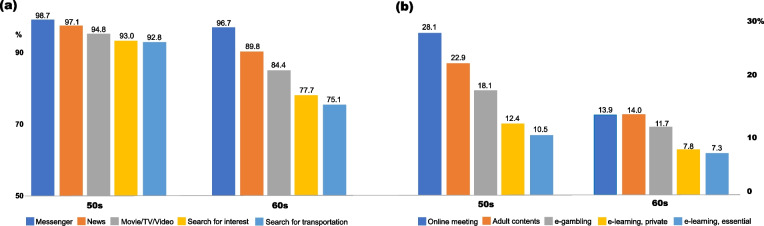
Top and bottom five rankings of the smartphone content used by participants

Figure 1 illustrates the top- and bottom-five rankings of the smartphone content used by participants. The most used content was “messenger,” “news,” and “movies/TV/videos,” whereas the least used content was “e-learning,” “e-gambling,” and “adult content.” The top- and bottom-five rankings were similar in both age groups.

**Fig. 2:**
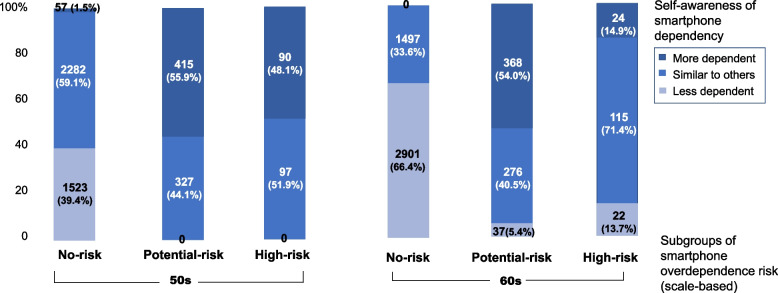
Categorization of smartphone overdependence risk by scale and self-awareness

Figure 2 compares the risk category based on the Smartphone Overdependence Scale and participants’ self-awareness of smartphone overdependence. Of the participants who were in their 50 s and at a high-risk of scale-based smartphone overdependence, 51.9% rated their smartphone overdependence as “similar to others” and 48.1% rated it as “more dependent than others.” Among the participants who were in their 60 s and at a high risk of scale-based smartphone overdependence, 13.7% rated their smartphone dependence as “less dependent than others” and 71.4% rated it as “similar to others.”

Table 2 presents the differences in participants’ demographics, smartphone content usage, digital literacy and psychosocial characteristics based on smartphone overdependence risk (no-risk *vs* potential-to-high risk). Among the participants in their 50 s, significant differences were observed based on educational levels (*p* = 0.006) and areas of residence (*p* < 0.001) according to the risk of smartphone overdependence. Additionally, digital literacy, social relations, and life satisfaction were significantly higher among individuals in the potential-to-high-risk group for smartphone overdependence (*p* ≤ 0.001) than in the no-risk group.

**Table 2:** Demographics, smartphone content usage, digital literacy and psychosocial characteristics based on the risk of smartphone overdependence

Variables	N (%) or Mean ± SD					
	50–59 years			60–69 years		
	No-risk (*N* = 3862)	Potential/high-risk (*N* = 929)	*p*-value^*^	No-risk (*N* = 4368)	Potential/high-risk (*N* = 842)	*p*-value^*^
**Sex**						
Male	1856 (48.1%)	439 (47.3%)	0.661	2097 (48.0%)	468 (53.6%)	< 0.001
Female	200 (51.9%)	490 (52.7%)		2271 (52.0%)	374 (44.4%)	
**Education**						
< middle school	66 (1.7%)	30 (3.2%)	0.006	1132 (25.9%)	179 (21.3%)	< 0.001
High school	2225 (57.6%)	546 (58.8%)		2805 (64.2%)	530 (62.9%)	
≥ University	1571 (40.7%)	353 (38.0%)		431 (9.9%)	133 (15.8%)	
**Monthly household income**						
< KRW 4 million	1056 (27.3%)	275 (29.6%)	0.218	2912 (66.7%)	519 (61.6%)	0.015
KRW 4–6 million	1747 (45.2%)	422 (45.4%)		860 (19.7%)	184 (21.9%)	
≥ KRW 6 million	1059 (27.4%)	232 (25.0%)		596 (13.6%)	139 (16.5%)	
**Job status**						
No	729 (18.9%)	183 (19.7%)	0.506	1406 (33.3%)	226 (26.8%)	< 0.001
Yes	3133 (81.1%)	746 (80.3%)		2902 (66.4%)	616 (73.2%)	
**Area of residence**						
Metropolitan	181 (46.9%)	424 (45.6%)	< 0.001	1729 (39.6%)	418 (49.6%)	< 0.001
City	1365 (35.2%)	287 (30.9%)		1502 (34.4%)	241 (28.6%)	
Small town	686 (17.8%)	218 (23.5%)		1137 (26.0%)	183 (21.7%)	
**Smartphone content used**						
Informative (6–42)	28.15 ± 6.32	28.17 ± 6.64	0.935	26.25 ± 7.44	26.73 ± 6.90	0.252
Entertainment (5–35)	21.23 ± 5.76	21.06 ± 6.40	0.673	19.30 ± 6.66	19.61 ± 7.00	0.527
Adult content/gambling (2–12)	4.83 ± 2.98	5.33 ± 3.27	0.071	3.99 ± 2.68	4.98 ± 3.06	0.001
Communication (4–24)	17.36 ± 4.02	17.41 ± 4.52	0.877	15.71 ± 4.84	16.33 ± 5.16	0.147
E-commerce (2–12)	8.52 ± 2.44	8.58 ± 2.59	0.587	7.81 ± 2.77	7.84 ± 2.79	0.859
Life management (3–21)	12.83 ± 3.37	12.81 ± 3.72	0.926	12.43 ± 3.78	12.27 ± 3.78	0.472
Work/education (2–12)	7.75 ± 4.19	8.47 ± 4.58	0.139	6.78 ± 4.69	6.93 ± 4.20	0.775
**Digital literacy** (6–24)	14.94 ± 3.59	15.51 ± 4.07	< 0.001	12.58 ± 4.10	13.90 ± 4.04	< 0.001
**Psychosocial characteristics**						
Social relations (3–12)	8.59 ± 1.61	8.79 ± 1.69	0.001	8.38 ± 1.67	8.36 ± 1.73	0.829
Life satisfaction (7–28)	20.80 ± 3.14	21.17 ± 3.21	0.001	20.09 ± 3.40	20.33 ± 3.24	0.063

^*^p-value from χ^2^ test or student’s t-test

Among the participants in their 60 s, significant differences were observed concerning sex (*p* < 0.001), educational level (*p* < 0.001), monthly household income (*p* = 0.015), job status (*p* < 0.001) and area of residence (*p* < 0.001) based on the risk of smartphone overdependence. Furthermore, the potential-to-high-risk group for smartphone overdependence exhibited significantly greater usage of adult content/gambling (*p* = 0.001) and demonstrated significantly higher digital literacy (*p* < 0.001) than the no-risk group. An additional file presents the differences in the score of each item [see Additional file 2].

Table 3 presents the ORs and corresponding 95% confidence intervals (CIs) of participants classified in the potential-to-high-risk group of smartphone overdependence. Individuals in their 50 s with a high school education exhibited a higher likelihood of being classified in the potential-to-high- risk group of smartphone overdependence, compared to those with an educational level above university (OR: 14.43 [1.49–139.68], *p* = 0.021). Furthermore, individuals with a job (OR: 0.38 [0.16–0.93], *p* = 0.033) and those frequently utilizing e-commerce-related smartphone contents exhibited significantly lower odds of potential-to-high risk (OR: 0.81 [0.70–0.94], *p* = 0.006].

**Table 3:** Factors associated with the potential-to-high risk for smartphone overdependence by age group

	50–59 years		60–69 years	
	OR [95% CI]	*p*-value^*^	OR [95% CI]	*p*-value^*^
**Female sex**	0.61 [0.29–1.29]	0.196	0.58 [0.28–1.19]	0.139
**Educational level** (Ref: University or higher)				
High school education	14.43 [1.49–139.68]	0.021	4.56 [1.08–19.31]	0.040
Middle school education or lower	1.50 [0.74–3.02]	0.263	2.26 [1.05–4.89]	0.038
**Monthly household income** (Ref: KRW < 4 million)				
KRW 4–6 million	1.62 [0.70–3.77]	0.263	1.34 [0.60–2.98]	0.472
KRW ≥ 6 million	0.80 [0.33–1.93]	0.619	1.05 [0.45–2.43]	0.915
**Having a job**	0.38 [0.16–0.93]	0.033	1.56 [0.71–3.40]	0.268
**Area of residence** (Ref: Metropolitan area)				
City	1.26 [0.56–2.86]	0.576	0.57 [0.24–1.36]	0.205
Small town	1.11 [0.46–2.64]	0.821	0.98 [0.44–2.16]	0.952
**Smartphone content usage**				
Informative content	1.06 [0.98–1.14]	0.147	1.00 [0.94–1.07]	0.946
Entertainment content	0.99 [0.92–1.08]	0.868	0.92 [0.85–1.00]	0.053
Adult content/gambling	1.127 [1.00–1.27]	0.058	1.27 [1.11–1.46]	0.001
Communication content	1.00 [0.90–1.12]	0.932	1.10 [0.99–1.23]	0.064
E-commerce content	0.81 [0.70–0.94]	0.006	0.98 [0.84–1.13]	0.739
Life management content	0.98 [0.88–1.09]	0.722	0.93 [0.83–1.03]	0.161
Work/education content	1.09 [0.98–1.21]	0.134	1.04 [0.93–1.16]	0.476
**Digital literacy**	1.11 [0.96–1.27]	0.161	1.07 [0.94–1.21]	0.294
**Social relations**	0.96 [0.77–1.19]	0.689	1.04 [0.81–1.33]	0.771
**Life satisfaction**	1.00 [0.88–1.13]	0.971	0.93 [0.80–1.07]	0.306

*Ref* Reference group, *OR* Odds ratio, *95% CI* 95% confidence interval

^*^p-value from multivariable binary logistic regression

Regarding individuals in their 60 s, those who had a high school education (OR: 4.56 [1.08–19.31], *p* = 0.040), had an educational level below middle school (OR: 2.26 [1.05–4.89], *p* = 0.038), and frequently engaged with adult content/gambling on smartphones (OR: 1.27 [1.11–1.46], *p* = 0.001) were significantly associated with higher odds of potential-to-high risk for smartphone overdependence.

## Discussion

This study revealed which content South Korean middle-aged and older adults use on their smartphones and to what extent they are at risk of smartphone overdependence. This study also highlighted the demographic, psychosocial, and smartphone-related factors that were associated with the potential-to-high risk of such overdependence.

The prevalence of smartphone overdependence among South Korean middle-aged and older adults has been increasing annually, driven by the surge in smartphone ownership [18]. This study found that in 2021, 14.2% and 3.5% of individuals aged 50 or older were at potential- and high-risk, respectively. These percentages—despite remaining lower than the corresponding rates in the total population (19.7% for potential- risk group and 4.5% for high risk) [16]—represent an increase of 47.6% among individuals in their 50 s and 49.6% among those in their 60 s, compared to the rates observed in 2016. This highlights the growing societal concerns regarding the risk of smartphone overdependence in this age group.

The results regarding participants’ smartphone content usage are interesting. In both age groups, greater than 95% of participants used messengers. Additionally, most of them used informative content, which corresponds to previous findings suggesting that older adults use smartphones more for non-social purposes than for social ones [36]. This study also found that the top and bottom five rankings were similar for both age groups. However, barring their use of messengers, older adults’ usage rate of the top five types of content was relatively low—less than 90%, whereas that of middle-aged adults exceeded 90%.

Regarding self-awareness of smartphone overdependence, older adults with high smartphone overdependence risk tended to have a lower awareness of their dependence than middle-aged adults in this study. Recognizing one’s addiction level is the first step toward preventing or overcoming addiction. Programs that educate individuals on the risk and prevention of smartphone overdependence would thus be beneficial for older adults.

This study identified several associated factors linked to smartphone overdependence among both age groups. Several studies have examined the effect of demographic factors and they have yielded differing results based on participants’ characteristics, the scales used, the presence of adjusted variables, and the type of adjustment [34, 37–40]. This study found that a lower educational level was significantly associated with a potential-to-high risk of smartphone overdependence among adults in both their 50 s and 60 s. Similar findings have been demonstrated in previous studies [34, 37], indicating a higher risk among individuals with lower educational levels. These findings emphasize the need for preventive measures tailored for individuals according to their educational backgrounds.

Regarding sex effects, some studies have found that females are more addicted to smartphones than males [38, 39], whereas other studies found no sex-based differences in the prevalence of smartphone overdependence [10, 40]. In the present study, the univariate analysis revealed that the proportion of males was significantly higher than females in the potential-to-high risk group of overdependence in individuals in their 60 s. However, this significant association disappeared after adjusting for variables in the multivariable analysis. Similarly, although participants’ risk differed significantly based on their areas of residence in both age groups when performing simple comparison, the multivariable analysis did not establish a significant association. These findings suggest a more complex interplay of confounding factors in determining the influence of demographic factors on smartphone overdependence.

We also examined the associations of smartphone overdependence with content usage patterns. Despite concerns regarding smartphones’ negative effects on mental health, they are commonly used for social networks and communication. In the digital era, individuals often experience the “fear of missing out or FOMO”—a pervasive apprehension that others might be having rewarding experiences from which one is absent—and a desire to stay connected with others [27]. Some previous studies have suggested undesirable effect of smartphones among extroverted and neurotic individuals who use social network services (SNSs) heavily [28]. In addition, a strong association was found between smartphone utilization for social purposes and smartphone overdependence [41, 42]. However, in other studies, the use of SNSs is positively correlated with life satisfaction and negatively correlated with depression and anxiety [26, 43]. Furthermore, individuals with high self-esteem and life satisfaction, even those who use SNSs heavily, do not experience problems arising from smartphone usage [44]. In this study, no significant association was found between smartphone use for communication-related content and the risk of overdependence, although messengers appeared to be the most frequently used content across both age groups.

Regarding other types of content utilization, frequent use for adult content/gambling was significantly associated with the potential-to-high risk of overdependence in individuals in their 60 s, which warrants special attention. Conversely, using the device for e-commerce was negatively associated with the potential-to-high risk among middle-aged adults.

Individuals with higher digital literacy are expected to use smartphones more frequently [23]. A previous study found that older adults’ smartphone usage increases their life satisfaction, and this relationship is mediated by digital literacy [23]. Another study indicated a positive relationship between digital literacy and smartphone overdependence: students, whose digital literacy increased with online learning experiences during the COVID-19 pandemic, also experienced an increase in nomophobia, or *no mo*bile phone *phobia* [24]. However, that study did not conduct multivariate analysis. This warrants a careful interpretation of its results because, in the present study, significant differences revealed by the univariable analysis disappeared after adjusting several variables in the multivariable analysis in both age groups.

Smartphone usage has been shown to have a double-edged sword effect on psychosocial factors. While it aggravates mental health, causing addiction, depression, and anxiety [10, 29], it also increases life satisfaction by reducing loneliness [26, 43]. In the survey used by this study, psychosocial factors were measured based on social relations and life satisfaction; however, no significant association of these factors with smartphone overdependence was identified in the multivariable analysis.

This study has several limitations, predominantly owing to the use of publicly available pre-existing data. First, the survey did not assess various additional psychological factors, such as depression and anxiety. The relationship between smartphone overdependence and psychological factors is multifaceted, and this area requires additional investigation. Second, the dataset did not include health-related information that could be associated with middle-aged and older adults’ problematic smartphone use. Future studies should examine the association between physical and mental health-related factors and smartphone overdependence among older adults. Third, the scales employed in the survey may have been influenced by the swiftly evolving digital landscape, warranting potential revision. Fourth, this study did not investigate and compare pre- and post-pandemic results.

## Conclusion

Despite the aforementioned limitations, this study highlighted the looming danger of smartphone overdependence among middle-aged and older adults. As this study was based on a large-scale nationwide survey, the results are representative of middle-aged and older adults in South Korea. The results will assist in developing of policies to prevent smartphone overdependence in middle-aged and older adults. Future studies on the health effects of excessive smartphone use are required for this age group.

### Supplementary Information

**Additional file 1.** Digital literacy, psychosocial characteristics, and smartphone dependence based on the age group, detailed. In the main text, the scores of digital literacy, social relations, life satisfaction, and the Smartphone Overdependence Scale are presented as categorical scores. However, this file indicates which item belonged to which category, the score for each item, and the age-group-based differences in each item.

**Additional file 2.** Differences in participants’ usage of smartphone content and their digital literacy and psychosocial factors based on their smartphone overdependence risk (no *versus *potential-to-high-risk), detailed. In the main text, the degree of smartphone content usages and the score of digital literacy, social relations, life satisfaction, and the Smartphone Overdependence Scale are presented as categorical scores (no-risk *versus* potential-to-high-risk). However, this file indicates which item belonged to which category and the score for each item, and the smartphone overdependence risk group-based differences in item among individuals in their 50s and 60s respectively (no *versus *potential-to-high-risk).

## Data Availability

The dataset supporting the conclusions of this article is openly available in the public data portal in South Korea, https://www.data.go.kr/data/15038425/fileData.do?recommendDataYn=Y.

## Abbreviations

DSM-5: Diagnostic and Statistical Manual of Mental Disorders-Fifth Edition
SD: Standard deviation
SNS: Social network service
