# Supplementary material for: Smartphone usage and overdependence risk among middle-aged and older adults: a cross-sectional study
Source: BMC Public Health. 2024 Feb 9;24:413. doi: 10.1186/s12889-024-17873-8 (PMC10854068; doi:10.1186/s12889-024-17873-8)
Supplement: Supplementary file 1 — Additional file 1. Digital literacy, psychosocial characteristics, and smartphone dependence based on the age group, detailed. In the main text, the scores of digital literacy, social relations, life satisfaction, and the Smartphone Overdependence Scale are presented as categorical scores. However, this file indicates which item belonged to which category, the score for each item, and the age-group-based differences in each item. [file 12889_2024_17873_MOESM1_ESM.docx]

Additional file 1. Digital literacy, psychosocial characteristics, and smartphone dependence based on the age group, detailed

In the main text, the scores of digital literacy, social relations, life satisfaction, and the Smartphone Overdependence Scale are presented as categorical scores. However, this file indicates which item belonged to which category, the score for each item, and the age-group-based differences in each item.

|  | | 50s | | 60s | |  |
| --- | --- | --- | --- | --- | --- | --- |
|  |  | Mean | SD | Mean | SD | P-value |
| Digital literacy^*^ | Ability to search information and content | 2.85 | 0.75 | 2.45 | 0.86 | 0.000 |
|  | Ability to assess reliable information online | 2.72 | 0.82 | 2.34 | 0.86 | 0.000 |
|  | Online social participation | 2.60 | 0.82 | 2.19 | 0.88 | 0.000 |
|  | Ability to produce and edit digital content | 2.08 | 0.90 | 1.74 | 0.85 | 0.000 |
|  | Privacy issue recognition | 2.41 | 0.87 | 2.06 | 0.88 | 0.000 |
|  | Using online information for academic or occupational activities | 2.39 | 0.91 | 2.02 | 0.91 | 0.000 |
| Social relation^*^ | Support from family | 3.00 | 0.75 | 2.92 | 0.77 | 0.000 |
|  | Support from friends | 2.96 | 0.67 | 2.86 | 0.68 | 0.000 |
|  | Support from society | 2.67 | 0.75 | 2.59 | 0.73 | 0.000 |
| Life satisfaction^*^ | Satisfaction with interpersonal relationship | 3.30 | 0.61 | 3.25 | 0.62 | 0.000 |
|  | Satisfaction with academic/occupational state | 3.04 | 0.70 | 2.91 | 0.71 | 0.000 |
|  | Satisfaction with health state | 3.07 | 0.74 | 2.84 | 0.76 | 0.000 |
|  | Satisfaction with consumption | 2.87 | 0.71 | 2.78 | 0.74 | 0.000 |
|  | Satisfaction with leisure | 2.75 | 0.75 | 2.69 | 0.77 | 0.000 |
|  | Satisfaction with social/economic achievement | 2.89 | 0.69 | 2.80 | 0.69 | 0.000 |
|  | Overall satisfaction | 2.95 | 0.64 | 2.87 | 0.63 | 0.000 |
| Smartphone Overdependence Scale^*^ | |  |  |  |  |  |
| Self-control failure | I attempt to spend less time using the smartphone; however, I always fail to do so. | 2.07 | 0.72 | 1.83 | 0.75 | 0.000 |
|  | It is difficult to control the time spent using the smartphone. | 2.05 | 0.77 | 1.81 | 0.80 | 0.000 |
|  | It is difficult to maintain the adequate time spent on smartphone use. | 2.05 | 0.78 | 1.83 | 0.80 | 0.000 |
| Salience | It is difficult to concentrate on other activities when the smartphone is within reach. | 1.92 | 0.76 | 1.75 | 0.78 | 0.000 |
|  | I cannot stop thinking about smartphones. | 1.85 | 0.77 | 1.67 | 0.78 | 0.000 |
|  | I have a strong urge to use my smartphone. | 1.85 | 0.81 | 1.67 | 0.79 | 0.000 |
|  | I have experienced health problems owing to smartphone use. | 1.78 | 0.78 | 1.66 | 0.78 | 0.000 |
| Problematic consequence | I have experienced conflict with a family member owing to smartphone use. | 1.65 | 0.73 | 1.59 | 0.74 | 0.000 |
|  | I have experienced severe conflicts with friends, colleagues, other social relationships owing to smartphone use. | 1.53 | 0.66 | 1.41 | 0.60 | 0.000 |
|  | I have difficulty performing my academic or occupational tasks owing to smartphone use. | 1.53 | 0.64 | 1.42 | 0.59 | 0.000 |
| Total | | 18.28 | 5.22 | 16.64 | 5.68 | 0.000 |

^*^4-point Likert scale (1 = not at all, 4 = very much). SD: standard deviation
