# Supplementary material for: Smartphone usage and overdependence risk among middle-aged and older adults: a cross-sectional study
Source: BMC Public Health. 2024 Feb 9;24:413. doi: 10.1186/s12889-024-17873-8 (PMC10854068; doi:10.1186/s12889-024-17873-8)
Supplement: Supplementary file 2 — Additional file 2. Differences in participants’ usage of smartphone content and their digital literacy and psychosocial factors based on their smartphone overdependence risk (no versus potential-to-high-risk), detailed. In the main text, the degree of smartphone content usages and the score of digital literacy, social relations, life satisfaction, and the Smartphone Overdependence Scale are presented as categorical scores (no-risk versus potential-to-high-risk). However, this file indicates which item belonged to which category and the score for each item, and the smartphone overdependence risk group-based differences in item among individuals in their 50s and 60s respectively (no versus potential-to-high-risk). [file 12889_2024_17873_MOESM2_ESM.docx]

Additional file 2. Differences in participants’ usage of smartphone content and their digital literacy and psychosocial factors based on their smartphone overdependence risk (no *versus* potential-to-high-risk), detailed

In the main text, the degree of smartphone content usages and the score of digital literacy, social relations, life satisfaction, and the Smartphone Overdependence Scale are presented as categorical scores (no-risk *versus* potential-to-high-risk). However, this file indicates which item belonged to which category and the score for each item, and the smartphone overdependence risk group-based differences in item among individuals in their 50s and 60s respectively (no *versus* potential-to-high-risk).

|  | 50s | | | | | 60s | | | | |
| --- | --- | --- | --- | --- | --- | --- | --- | --- | --- | --- |
|  | No-risk | | Potential/high-risk | |  | No-risk | | Potential/high-risk | |  |
| **Smartphone contents^*^** | Mean | SD | Mean | SD | p-value | Mean | SD | Mean | SD | p-value |
| News | 4.97 | 1.47 | 5.09 | 1.42 | .021 | 4.71 | 1.54 | 5.01 | 1.40 | <.001 |
| Searches for study/work | 4.35 | 1.65 | 4.44 | 1.7 | .181 | 3.77 | 1.75 | 4.07 | 1.77 | 0.001 |
| Searches for hobby | 4.75 | 1.29 | 4.76 | 1.35 | .884 | 4.33 | 1.44 | 4.69 | 1.22 | <.001 |
| Searches for goods/service | 4.73 | 1.33 | 4.77 | 1.32 | .450 | 4.37 | 1.47 | 4.47 | 1.39 | .118 |
| Searches for transportation | 4.57 | 1.41 | 4.7 | 1.44 | .013 | 4.37 | 1.53 | 4.44 | 1.41 | .218 |
| Other web-surfing | 4.23 | 1.46 | 4.22 | 1.55 | .943 | 3.89 | 1.51 | 4.01 | 1.50 | .109 |
| Games | 4.22 | 1.65 | 4.26 | 1.66 | .660 | 4.00 | 1.69 | 4.25 | 1.60 | .001 |
| Movies/television/videos (e.g., YouTube, Netflix) | 5.20 | 1.34 | 5.19 | 1.34 | .825 | 4.97 | 1.47 | 5.09 | 1.46 | .050 |
| Music | 4.67 | 1.48 | 4.82 | 1.48 | .007 | 4.57 | 1.56 | 4.59 | 1.53 | .755 |
| Radio, podcasts | 3.71 | 1.65 | 3.98 | 1.74 | .002 | 3.63 | 1.83 | 3.73 | 1.57 | .245 |
| E-book, webtoons, web stories | 3.59 | 1.72 | 3.64 | 1.72 | .610 | 3.07 | 1.77 | 3.35 | 1.79 | .031 |
| Adults content | 2.57 | 1.55 | 2.86 | 1.72 | .021 | 2.24 | 1.49 | 2.63 | 1.61 | .003 |
| Gambling games (e.g., sports betting, online gambling) | 2.36 | 1.50 | 2.62 | 1.70 | .059 | 1.95 | 1.37 | 2.48 | 1.55 | <.001 |
| E-mail | 3.92 | 1.57 | 3.84 | 1.57 | .257 | 3.43 | 1.60 | 3.78 | 1.68 |  |
| Messenger | 5.55 | 1.30 | 5.52 | 1.28 | .541 | 5.24 | 1.44 | 5.52 | 1.31 | <.001 |
| Social network services | 4.80 | 1.43 | 4.89 | 1.52 | .179 | 4.52 | 1.58 | 4.72 | 1.65 | .008 |
| Dating and meeting new friends | 3.03 | 1.56 | 3.30 | 1.64 | .010 | 2.82 | 1.66 | 2.97 | 1.62 | .214 |
| Buying goods | 4.48 | 1.34 | 4.48 | 1.37 | .941 | 4.12 | 1.45 | 4.18 | 1.47 | .414 |
| Selling goods | 3.94 | 1.48 | 4.03 | 1.51 | .213 | 3.63 | 1.59 | 3.74 | 1.56 | .242 |
| Finance | 4.69 | 1.34 | 4.70 | 1.36 | .898 | 4.33 | 1.42 | 4.51 | 1.43 | .006 |
| Life | 3.93 | 1.47 | 3.93 | 1.59 | .982 | 3.85 | 1.52 | 3.87 | 1.51 | .779 |
| Health | 4.07 | 1.47 | 4.12 | 1.51 | .431 | 4.24 | 1.56 | 3.98 | 1.50 | .001 |
| Online meeting/remote work | 3.45 | 1.59 | 3.50 | 1.62 | .646 | 2.89 | 1.72 | 3.05 | 1.62 | .278 |
| E-learning, essential | 2.59 | 1.65 | 2.77 | 1.8 | .321 | 2.24 | 1.66 | 2.21 | 1.43 | .887 |
| E-learning, private | 2.76 | 1.69 | 2.91 | 1.78 | .384 | 2.30 | 1.71 | 2.35 | 1.68 | .796 |
| **Digital literacy^**^** |  |  |  |  |  |  |  |  |  |  |
| Ability to search information and content | 2.82 | 0.75 | 2.98 | 0.77 | <.001 | 2.42 | 0.86 | 2.65 | 0.85 | <.001 |
| Ability to assess reliable information online | 2.70 | 0.81 | 2.79 | 0.84 | .003 | 2.31 | 0.86 | 2.47 | 0.85 | <.001 |
| Online social participation | 2.59 | 0.81 | 2.64 | 0.88 | .177 | 2.15 | 0.87 | 2.38 | 0.89 | <.001 |
| Ability to produce and edit digital content | 2.06 | 0.88 | 2.18 | 0.98 | <.001 | 1.70 | 0.83 | 1.93 | 0.93 | <.001 |
| Privacy issue recognition | 2.39 | 0.85 | 2.47 | 0.94 | .021 | 2.02 | 0.87 | 2.25 | 0.88 | <.001 |
| Using online information for academic or occupational activities | 2.37 | 0.89 | 2.45 | 0.98 | .030 | 1.98 | 0.90 | 2.22 | 0.93 | <.001 |
| **Social relation^**^** |  |  |  |  |  |  |  |  |  |  |
| Support from family | 2.99 | 0.75 | 3.07 | 0.76 | .002 | 2.93 | 0.76 | 2.89 | 0.79 | .160 |
| Support from friends | 2.96 | 0.67 | 2.96 | 0.68 | .888 | 2.87 | 0.67 | 2.83 | 0.69 | .139 |
| Support from society | 2.64 | 0.73 | 2.76 | 0.78 | <.001 | 2.58 | 0.73 | 2.65 | 0.73 | .015 |
| **Life satisfaction^**^** |  |  |  |  |  |  |  |  |  |  |
| Satisfaction with interpersonal relationship | 3.28 | 0.61 | 3.37 | 0.62 | <.001 | 3.25 | 0.61 | 3.22 | 0.64 | .215 |
| Satisfaction with academic/occupational state | 3.03 | 0.70 | 3.08 | 0.71 | .063 | 2.90 | 0.71 | 2.96 | 0.68 | .024 |
| Satisfaction with health state | 3.08 | 0.74 | 3.05 | 0.73 | .442 | 2.83 | 0.76 | 2.88 | 0.74 | .111 |
| Satisfaction with consumption | 2.86 | 0.71 | 2.94 | 0.72 | .002 | 2.77 | 0.74 | 2.83 | 0.71 | .037 |
| Satisfaction with leisure | 2.73 | 0.75 | 2.84 | 0.76 | <.001 | 2.68 | 0.77 | 2.74 | 0.76 | .025 |
| Satisfaction with social/economic achievement | 2.88 | 0.68 | 2.92 | 0.71 | .121 | 2.79 | 0.69 | 2.81 | 0.70 | .467 |
| Overall satisfaction | 2.94 | 0.63 | 2.97 | 0.67 | .154 | 2.87 | 0.62 | 2.89 | 0.67 | .404 |

**^*^** 7-point Likert scale (1 = rarely, 7 = very frequently), **^**^** 4-point Likert scale (1 = not at all, 4: very much)

SD: standard deviation
